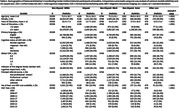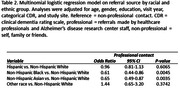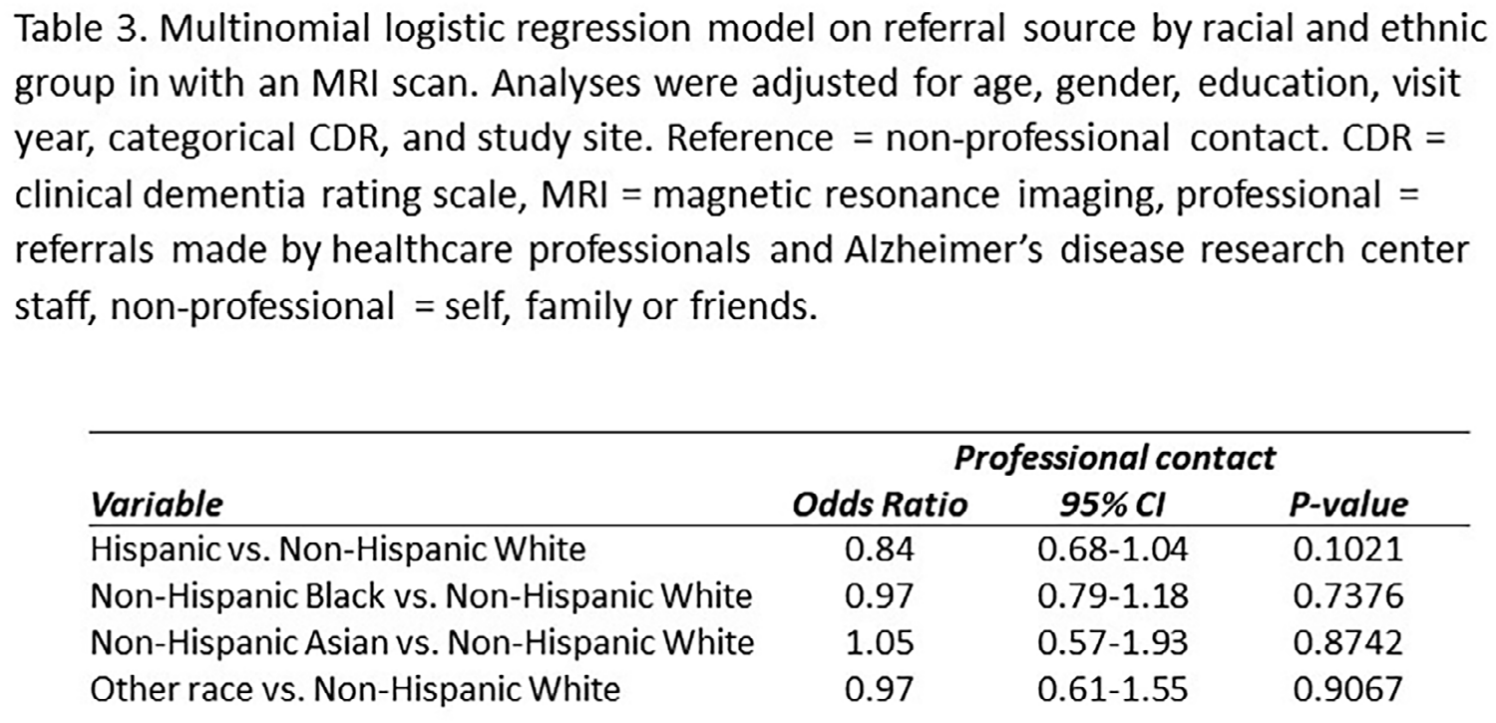# Differences in referral source across racial and ethnic groups at Alzheimer’s Disease Research Centers

**DOI:** 10.1002/alz.085999

**Published:** 2025-01-09

**Authors:** Carol Chan, Kathleen A. Lane, Sujuan Gao, Omolola A Adeoye‐Olatunde, Sarah A Biber, Shannon L. Risacher, Andrew J. Saykin, Sophia Wang

**Affiliations:** ^1^ Case Western Reserve University School of Medicine, Cleveland, OH USA; ^2^ Indiana University School of Medicine, Indianapolis, IN USA; ^3^ Indiana Alzheimer’s Disease Research Center, Indianapolis, IN USA; ^4^ Purdue University College of Pharmacy, West Lafayette, IN USA; ^5^ University of Washington, Seattle, WA USA; ^6^ National Alzheimer’s Coordinating Center, University of Washington, Seattle, WA USA; ^7^ Indiana University, Indianapolis, IN USA

## Abstract

**Background:**

Despite recognition of the need to increase underrepresented groups (URG) engagement in Alzheimer’s disease and related dementias (ADRD) studies, enrollment remains low. As a first step in examining these disparities, these analyses aimed to compare referral sources for Alzheimer’s Disease Research Centers (ADRC) enrollment of URG participants.

**Method:**

These analyses included data from 48,330 participants across 46 ADRCs, obtained through the National Alzheimer’s Coordinating Center Uniform Data Set. Generalized logistic regression models with generalized estimating equations were used to examine the association of racial/ethnic group and professional vs non‐professional referral source. The ‘professional’ category included referrals made by healthcare professionals or ADRC staff, while the ‘non‐professional’ category included referrals made by self, family or friends. This association was examined across the entire sample, and then individuals who had completed magnetic resonance imaging (MRI). The analyses were adjusted for age, gender, education, visit year, and categorical CDR with random site effect to adjust for study site.

**Result:**

Descriptive statistics are shown in Table 1. Non‐Hispanic Black and Asian participants were less likely to have completed an MRI. Across the entire sample, Non‐Hispanic Black and Non‐Hispanic Asian participants were less likely to be referred by a professional contact than Non‐Hispanic White participants (Table 2). In those who had completed an MRI, there were no significant differences across the racial groups, although we note that the sample sizes for those with MRI were much smaller (Table 3). Results for both analyses were similar when only participants who had a diagnosis of MCI or dementia and a global CDR of 0.5 or 1 at baseline were included.

**Conclusion:**

One major factor leading to lower rates of URG participation in ADRD research is disproportionately fewer healthcare professional referrals. To develop and optimize ADRC recruitment strategies, future studies are needed to explore reasons for differences in URG referrals by healthcare professionals and non‐professionals.